# Lower Serum Caveolin-1 Is Associated with Cerebral Microbleeds in Patients with Acute Ischemic Stroke

**DOI:** 10.1155/2016/9026787

**Published:** 2016-03-28

**Authors:** Jun Zhang, Wusheng Zhu, Lulu Xiao, Qinqin Cao, Hao Zhang, Huaiming Wang, Zusen Ye, Yonggang Hao, Qiliang Dai, Wen Sun, Yunyun Xiong, Xinfeng Liu, Ruidong Ye, Gelin Xu

**Affiliations:** ^1^Department of Neurology, Jinling Hospital, Medical School of Nanjing University, Nanjing, Jiangsu 210002, China; ^2^Department of Neurology, Jinling Hospital, Second Military Medical University, Nanjing, Jiangsu 210002, China; ^3^Department of Neurology, Jinling Hospital, Southern Medical University, Nanjing, Jiangsu 210002, China

## Abstract

Caveolin-1 (Cav-1) plays pivotal roles in the endothelial damage following stroke. The present study aimed to investigate whether serum Cav-1 level is associated with the presence of cerebral small vessel disease (cSVD) in patients with acute ischemic stroke. To this end, 156 patients were consecutively enrolled. Cranial magnetic resonance imaging was analyzed to determine the surrogates of cSVD, including cerebral microbleeds (CMBs), silent lacunar infarcts (SLIs), and white matter hyperintensities (WMHs). After adjusting for potential confounders, patients with low Cav-1 level had a higher risk of CMBs than patients with high Cav-1 level (OR: 4.05, 95% CI: 1.77–9.30). However, there was no relationship between Cav-1 and the presence of SLIs or WMHs. When CMBs were stratified by location and number, a similar association was found in patients with deep or infratentorial CMBs (OR: 4.04, 95% CI: 1.59–10.25) and with multiple CMBs (OR: 3.18, 95% CI: 1.16–8.72). These results suggest lower serum Cav-1 levels may be associated with CMBs, especially those that are multiple and located in deep brain or infratentorial structures, in patients with acute ischemic stroke. Cav-1 may be involved in the pathophysiology of CMBs, and may act as a potential target for treating cSVD.

## 1. Introduction

Caveolae are small flask-shaped invaginations of the cell plasma membrane and are particularly abundant in endothelial cells. They participate in many intra- and intercellular activities, such as endocytosis, vesicular trafficking, and signal transduction [1, 2]. As a key structural protein of caveolae domains, caveolin-1 (Cav-1) participates in regulating blood-brain barrier (BBB) permeability, oxidative stress, and counteracting neuroinflammatory process [3, 4]. In laboratory stroke investigations, Cav-1 levels have been associated with neuronal apoptosis, BBB disruption, infarction enlargement, and functional deterioration [5, 6], yet there has been no clinical research to confirm these relationships to date.

Cerebral small vessel disease (cSVD) is mainly characterized by cerebral microbleeds (CMBs), silent lacunar infarcts (SLIs), and white matter hyperintensities (WMHs) in magnetic resonance imaging (MRI) [7]. These trinity imaging surrogates have been associated with increased risk of subsequent cerebrovascular events and unfavorable functional outcomes in ischemic stroke survivors [8–12]. Although potential unknown mechanisms are thought to participate in the development of cSVD, previous studies demonstrated that inflammatory cascade activated by endothelial lesion and BBB damage is involved in the initiation and progression of cSVD [7, 13]. Considering a salient role of Cav-1 in these pathophysiologic processes, we hypothesized that Cav-1 levels might be related to the presence of cSVD. Therefore, in this study, we investigated the association between serum Cav-1 levels and cSVD detected and measured with MRI in a cohort of patients with ischemic stroke.

## 2. Subjects and Methods

### 2.1. Study Subjects

From January 2013 to December 2014, patients with ischemic stroke registered in Nanjing Stroke Registry Program (NSRP) were prospectively enrolled. NSRP has been described previously in detail [14]. Patients were enrolled in this study if they are (1) with first-ever ischemic stroke within 24 hours of symptom onset and (2) aged 18 years or older. We excluded patients if they are (1) with traumatic brain injury or brain surgery, (2) with chronic kidney diseases, (3) with malignant tumor, and (4) currently with infectious disease. All patients provided informed consent, and the study was approved by the ethics committee of Jinling Hospital.

According to the inclusion criteria, there were 172 patients with first-ever ischemic stroke within 24 hours of symptom onset and 18 years or older. The ischemic stroke was confirmed by professional neurologist with acute stroke syndrome and subsequent imaging examination. During this period, 7 patients refused to participate. Additionally, 2 patients with traumatic brain injury or brain surgery, 2 with chronic kidney disease, 2 with malignant tumor, and 3 with infectious disease were excluded. Accordingly, a total of 156 patients were included in final analysis.

### 2.2. MRI Protocols

Cranial MRI imaging was performed with a 3.0 T Trio MRI scanner (Siemens, Erlangen, Germany) with a 12-channel head coil. The imaging protocol consisted of axial T1-weighted, T2-weighted, fluid-attenuated inversion recovery (FLAIR), diffusion-weighted imaging (DWI), and susceptibility-weighted imaging (SWI) sequences. T1-weighted, T2-weighted, FLAIR, and DWI sequences were performed according to previously standardized procedures and parameters: T1-weighted: repetition time (TR), 350 ms; echo time (TE), 2.46 ms; T2-weighted: TR, 4000 ms; TE, 98 ms; T2-FLAIR: TR, 7000 ms; TE, 87 ms; DWI: TR, 3000 ms; TE, 91 ms. SWI was performed with the following parameters: matrix size, 512 × 254 × 72; field of view (FOV), 230 × 115 × 144 mm^3^; TR, 56 ms; TE, 25 ms; flip angle, 20°. The detail has been described in our previous study [15].

### 2.3. Cranial MRI Surrogates of cSVD

The MRI surrogates of cSVD of interest were CMBs, SLIs, and WMHs. Imaging results from all included patients (*n* = 156) were assessed by 2 raters who were blind to the clinical information. Disagreements were resolved by consultation with a third reviewer. CMBs were defined as small round hypointensity areas (2–10 mm in diameter) on SWI sequence [16, 17] and were categorized according to their locations as lobar (cortical gray and subcortical or periventricular white matter), deep (deep gray matter: basal ganglia and thalamus, and the white matter of the corpus callosum, internal, eternal, and extreme capsule), and infratentorial (brainstem and cerebellum). Furthermore, patients with CMBs were dichotomized according to the presence or absence of microbleeds in deep or infratentorial locations. Those with microbleeds in deep or infratentorial locations were defined as “deep or infratentorial microbleeds” and those without were defined as “strictly lobar microbleeds” [18]. CMBs were further categorized according to their numbers as absent, single (1 CMB), and multiple (≥2 CMBs). CMBs mimics, such as calcifications, iron deposits, and flow voids in pial blood vessel were carefully excluded [16, 17]. Interrater reliability for the presence of CMBs was 0.85.

SLIs were defined as focal lesions (3–15 mm in diameter), accompanied by hypointensity on T1 image, corresponding hyperintensity on T2 image, and hypointensity with perifocal high signal on T2 FLAIR image [19]. WMHs were defined as hyperintensity surrounding the ventricles and in the deep white matter on FLARI images, classified by a modified Fazekas rating scale. Periventricular hyperintensity was graded as 0 = absence, 1 = “caps” or pencil-thin lining, 2 = smooth “halo,” or 3 = irregular extending into the deep white matter. Deep white matter hyperintensity was rated as 0 = absence, 1 = punctate foci, 2 = beginning confluence of foci, or 3 = large confluent areas [20]. The presence of WMHs was defined when grade is ≥1 of any location. Interrater reliability for the presence of SLIs and WMHs was 0.78 and 0.81, respectively.

### 2.4. Cerebrovascular Risk Factors

Baseline characteristics of patients were collected at the time of admission. Hypertension was defined as systolic blood pressure (SBP) ≥ 140 mmHg, diastolic blood pressure (DBP) ≥ 90 mmHg, or use of antihypertensive medications. Diabetes mellitus was diagnosed if either the fasting glucose level was ≥126 mg/dL or the participants were currently being treated with antidiabetic agents. Hyperlipidemia was defined as an elevated level of triglycerides (≥150 mg/dL), total cholesterol (≥220 mg/dL), or low-density lipoprotein cholesterol (≥140 mg/dL) or having received lipid-lowering drugs. Coronary heart disease, atrial fibrillation, and myocardial infarction were all recognized as heart disease. Body mass index (BMI) was calculated as weight (kg)/height (m^2^). Smoking was defined as currently smoking or having quit for ≤2 years. Drinking was defined as current alcohol drinking ≥20 g/d.

### 2.5. Serum Cav-1 Measurement

Morning blood samples for measurements of glucose, lipid, fibrinogen, and Cav-1 levels were obtained after an overnight fast within 48 hours of symptom onset. Blood was centrifuged at 1500 g for 10 minutes within 30 minutes of collection, and the serum was stored at −80°C. Cav-1 levels were measured by commercially available enzyme-linked immunosorbent assay kit (Uscn Life Science, Wuhan, China) according to the manufacturer's instructions. The intra-assay coefficient was <10%.

### 2.6. Statistical Analysis

Continuous variables were expressed as mean ± SD or median (interquartile range) and compared with Student's *t*-test or Mann-Whitney *U* test. Categorical variables were expressed as percentages and compared using Chi-square or Fisher's exact test. Logistic regression was used to evaluate the relationship between Cav-1 and cSVD by calculating adjusted odds ratios (OR) and 95% confidence intervals (CI). Cav-1 levels were divided into dichotomy with cut points of 5.25 ng/mL. Variables at a level of *P* < 0.1 in univariable comparison and those being reported previously as potential confounders were adjusted in multivariable logistic regression, which were first adjusted for age and sex (Model 1) and then for all potential confounders (Model 2). A two-tailed *α* value of 0.05 was deemed statistically significant. All data analyses were performed using SPSS 19.0.

## 3. Results

### 3.1. Baseline Characteristics

A total of 156 patients (68.6%, male; mean age, 63.2 ± 9.1 years) were enrolled in the study. The baseline characteristics of the patients were presented in Table 1. Overall, 104 (66.7%) patients had hypertension, 37 (23.7%) patients had diabetes mellitus, 52 (33.3%) patients had hyperlipidemia, and 22 (14.1%) patients had heart disease. The mean ± SD (range) Cav-1 level of these patients was 5.62 ± 2.63 (1.67–12.32) ng/mL.

**Figure 1:**
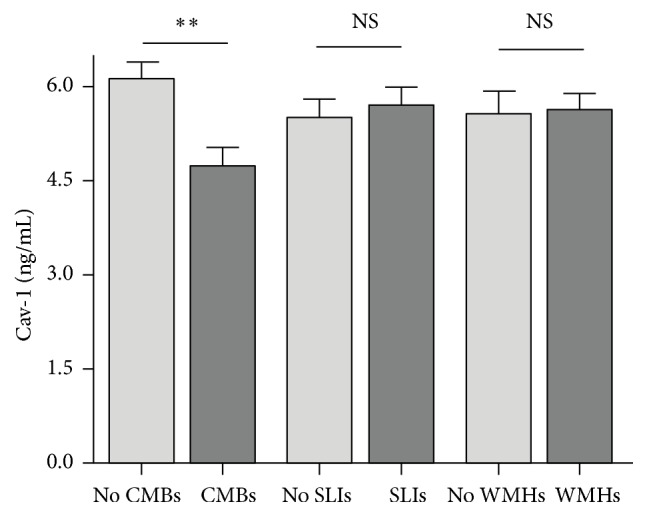
Mean ± SEM of Cav-1 levels are shown according to the presence of cSVD. ^*∗∗*^ *P* < 0.01 compared with corresponding negative group. CMBs indicate cerebral microbleeds. SLIs indicate silent lacunar infarcts. WMHs indicate white matter hyperintensities. Cav-1 indicates caveolin-1.

Based on MRI results, there were 57 (36.5%) patients with CMBs, 82 (52.6%) patients with SLIs, and 95 (60.9%) patients with WMHs. Univariable comparison showed that patients with CMBs were older than patients without (65.1 ± 8.4 versus 62.1 ± 9.4, *P* = 0.046). Patients with CMBs had higher incidence of hypertension (77.2% versus 60.6%, *P* = 0.034) and higher systolic blood pressure (140 versus 135 mmHg, *P* = 0.013) at admission. The levels of blood fibrinogen were higher (310.9 ± 61.1 versus 287.6 ± 57.1 mg/dL, *P* = 0.018) in patients with CMBs than in patients without. Patients with CMBs had lower serum Cav-1 levels (4.74 ± 2.26 versus 6.12 ± 2.71 ng/mL, *P* = 0.001) than patients without. For ischemic cSVD, patients with SLIs were older than patients without (65.0 ± 8.7 versus 61.2 ± 9.2, *P* = 0.009). Systolic blood pressure at admission was higher in patients with SLIs (140 versus 137 mmHg, *P* = 0.029) than in patients without. Similarly, patients with WMHs were older than patients without (64.7 ± 9.0 versus 60.9 ± 8.9, *P* = 0.011). And patients with WMHs had higher prevalence of hypertension (73.7% versus 55.7%, *P* = 0.020) and higher systolic blood pressure at admission (140 versus 134 mmHg, *P* = 0.015) than patients without. However, there were no significant differences concerning the levels of serum Cav-1 between patients with SLIs and patients without (5.71 ± 2.77 versus 5.51 ± 2.49 ng/mL, *P* = 0.634) or between patients with WMHs and patients without (5.65 ± 2.58 versus 5.58 ± 2.74 ng/mL, *P* = 0.875, Figure 1).

### 3.2. Relationship between Cav-1 and cSVD

After adjusting for age and sex (model 1), presence of CMBs in patients with low Cav-1 level (≤5.25 ng/mL) was higher (OR = 3.69, 95% CI 1.78–7.64, *P* = 0.00044) than that in patients with high Cav-1 level (>5.25 ng/mL). After adjusting for more confounders (model 2), presence of CMBs was still higher in patients with low Cav-1 level (OR = 4.05, 95% CI 1.77–9.30, *P* = 0.001) than that in patients with high Cav-1 level. However, there were no significant differences between Cav-1 and SLIs (OR = 1.61, 95% CI 0.77–3.36, *P* = 0.209, Model 2) or WMHs (OR = 0.77, 95% CI 0.36–1.67, *P* = 0.510, Model 2), respectively (Table 2).

### 3.3. Relationship between Cav-1 and CMBs Subgroups

**Figure 2:**
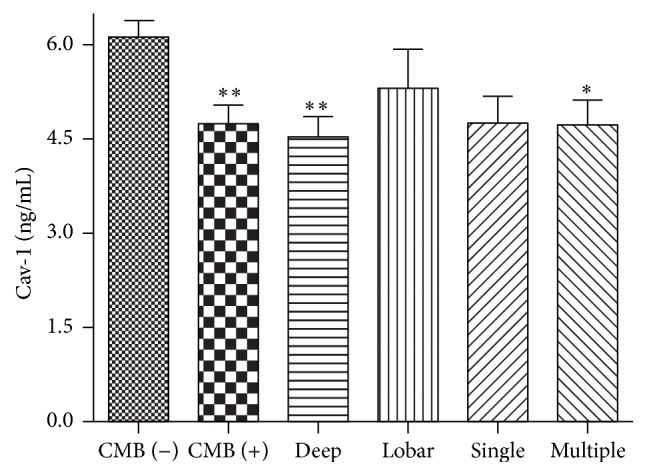
Mean ± SEM of Cav-1 levels are shown according to the presence, location, and number of CMBs. ^*∗*^ *P* < 0.05; ^*∗∗*^ *P* < 0.01 compared with CMBs (−) group. CMBs indicate cerebral microbleeds. Cav-1 indicates caveolin-1.

Of the 57 patients with CMBs, 22 (38.6%) were categorized as being with single CMBs and 35 (61.4%) as being with multiple CMBs. CMBs were more frequently observed in deep brain or infratentorial structures (42/57, 73.7%) than in lobar areas (15/57, 26.3%). When CMBs were categorized according to their locations, patients with CMBs in deep brain or infratentorial structures had significantly lower levels of Cav-1 than patients without CMBs (4.53 ± 2.22 versus 6.12 ± 2.71 ng/mL, *P* = 0.003). However, there was no significant difference concerning Cav-l levels between patients with lobar CMBs and patients without CMBs (5.32 ± 2.37 versus 6.12 ± 2.71 ng/mL, *P* = 0.764). When CMBs were categorized according to their numbers, patients with multiple CMBs had lower levels of Cav-1 than patients without CMBs (4.72 ± 2.43 versus 6.12 ± 2.71 ng/mL, *P* = 0.019, Figure 2).

Logistic regression analysis showed that the presence of deep or infratentorial CMBs was higher in patients with low Cav-1 levels than that in patients with high Cav-1 level (OR = 3.54, 95% CI 1.58–7.92, *P* = 0.002, Model 1). After adjusting for more confounders, the association still existed (OR = 4.04, 95% CI 1.59–10.25, *P* = 0.003, Model 2). Similar association was found between Cav-1 and the presence of multiple CMBs (OR = 3.18, 95% CI 1.16–8.72, *P* = 0.025, Model 2). However, no association was found between Cav-1 and strictly lobar CMBs (OR = 1.55, 95% CI 0.43–5.60, *P* = 0.501, Model 2) or single CMBs (OR = 3.03, 95% CI 0.95–9.72, *P* = 0.062, Model 2), respectively (Table 3).

## 4. Discussion

This study found that lower serum Cav-1 level is associated with the presence of CMBs in acute ischemic stroke. After adjusting for potential confounders, patients with low Cav-1 level had a 3-fold increased risk of CMBs compared with patients with high Cav-1 level. When CMBs were categorized according to number and location, the low serum Cav-1 levels were independently associated with multiple CMBs and deep or infratentorial CMBs.

Growing evidence has suggested that Cav-1 is involved in the regulation of lipoprotein transcytosis across endothelial cells and in the regulation of vascular inflammation and mitochondrial oxidative metabolism. Cav-1 knockout (KO) ischemic stroke models exhibited increased BBB permeability, redox imbalance, and amplified proinflammatory cytokines [3, 21]. When Cav-1 was downregulated, there was an increase of matrix metalloproteinases- (MMP-) 2/9 activity, which can hydrolyze BBB extracellular matrix and tight junction (TJ) proteins and subsequently leads to the BBB opening [22]. As such, decreased Cav-1 expression can increase the activation of endothelial nitric oxide synthase (eNOS), which increases NO production in endothelial cells and leads to remarkably endothelial and microvascular hyperpermeability [23, 24]. On the other hand, downregulation of Cav-1 was reported to have amplified proinflammatory cytokines, including IL-1*β*, IL-2, IL-6, and IL-9 [3, 19]. Deficiency of Cav-1 presented altered redox homeostasis and promoted a significant increase of oxidative stress in endothelial cell, which possibly reflects a role of Cav-1 in mitochondrial function [25]. These molecular mechanisms are also supposed to be associated with the occurrence of cSVD. As found in this clinical study, lower serum Cav-1 levels were associated with the presence of CMBs.

CMBs, as a novel image surrogate of cSVD, have been proved to be associated with endothelial dysfunction, BBB leakage, inflammation activation, and oxidative stress [7, 13, 19, 26]. Histopathological studies demonstrate that the spatial distribution of CMBs may reflect specific underlying vascular pathological changes, in particular cerebral amyloid angiopathy (CAA) and hypertensive vasculopathy [17]. These two disorders are characterized by different patterns of microbleeds distribution: hypertensive vasculopathy is usually associated with CMBs in the basal ganglia, thalamus, brainstem, and cerebellum [27], whereas CAA is associated with lobar distribution [28]. In this study, when CMBs were categorized according to their locations, the lower serum Cav-1 levels were independently associated with deep or infratentorial CMBs but not with lobar CMBs. These findings might partly indicate that Cav-1 may be involved in the pathogenesis of hypertensive vasculopathy rather than CAA. Recent studies also have revealed that Cav-1 was associated with pulmonary arterial hypertension and was upregulated in high-salt diet-induced endothelial dysfunction and hypertension in type 1 diabetes [29, 30]. However, no evidence demonstrated that there was a relationship between Cav-1 and CAA-related pathology [31], which may partly explain our results.

For ischemic cSVD, there were no remarkable associations between serum Cav-1 levels and SLIs or WMHs in this study. When we repeated the analysis for WMHs with Fazekas 0-1 versus Fazekas 2-3, there was still no significant association (OR = 1.55, 95% CI 0.69–3.46, *P* = 0.286, Model 2). Although CMBs, SLIs, and WMHs are common and representative surrogates of cSVD, there might be differences in risk factors and specific pathophysiology between ischemic and hemorrhagic cSVD [13, 32, 33]. CMBs are more likely to reflect BBB disruption and induced oxidative stress and chronic inflammation, while SILs and WMHs are more likely to be associated with ischemic damage of small perforating arterial or emboli. Some inflammatory biomarker profiles were observed with different levels between hemorrhagic and ischemic MRI surrogates of cSVD [7]. As a plasma membrane cell protein, Cav-1 plays a significant role in regulating endothelial function and BBB permeability. Other experiments revealed that deficiency of Cav-1 appeared atheroprotective and decreased plague area. This phenotype was attributed mainly to defective transendothelial migration of low-density lipoprotein [34, 35]. In the current study, although the levels of serum Cav-1 were higher in SLIs and WMHs groups than those in corresponding negative group of each surrogate, there were no significant differences concerning the levels of serum Cav-1 between patients with SLIs and patients without SLIs or between patients with WMHs and patients without WMHs. This finding deserves confirmation by further studies.

There are limitations which should be emphasized when interpreting the results. First, the sample size is relatively small, which might jeopardize the power of the study, especially when evaluating the effects of CMBs subgroups. Second, blood samples were obtained at one time point within 48 hours of symptom onset. A serial observation of the dynamic changes of Cav-1 levels is lacking. Third, in addition to these typically MRI surrogates, enlarged perivascular space and brain atrophy have been recently suggested as constituents of cSVD, which also deserve further study.

In conclusion, lower serum Cav-1 levels may be associated with CMBs, especially those that are multiple or located in deep brain or infratentorial structures, in patients with acute ischemic stroke. Before being generalized to other populations, these results warrant further studies to establish Cav-1 as a biomarker for predicting CMBs. Observational studies focusing on elderly without stroke and interventional studies in animals (such as gene knockout studies) are needed to determine whether Cav-1 is really a marker of CMBs and a potential target for treating cerebral small vessel disease.

## Figures and Tables

**Table 1:** Baseline characteristics of patients according to the presence of cSVD.

Variables	All patients *n* = 156	CMBs (+) *n* = 57	SLIs (+) *n* = 82	WMHs (+) *n* = 95
Age, y	63.2 ± 9.1	65.1 ± 8.4^**∗**^	65.0 ± 8.7^**∗∗**^	64.7 ± 9.0^**∗**^
Male, %	107 (68.6)	44 (77.2)	60 (73.2)	62 (65.3)
Hypertension, %	104 (66.7)	44 (77.2)^**∗**^	56 (68.3)	70 (73.7)^**∗**^
Diabetes mellitus, %	37 (23.7)	16 (28.1)	22 (26.8)	23 (24.2)
Dyslipidemia, %	52 (33.3)	15 (26.3)	25 (30.5)	30 (31.6)
Heart disease, %	22 (14.1)	8 (14.0)	14 (17.1)	13 (13.7)
BMI, kg/m^2^	25.1 (23.9–26.0)	24.9 (24.0–25.9)	25.3 (23.9–26.1)	24.9 (23.9–25.8)
Smoking, %	54 (34.6)	19 (33.3)	28 (34.1)	35 (36.8)
Alcohol intake, %	59 (37.8)	22 (38.6)	33 (40.2)	38 (40.0)
SBP, mmHg	140 (130–145)	140 (131–149)^**∗**^	140 (130–148)^**∗**^	140 (130–148)^**∗**^
DBP, mmHg	85 (80–90)	90 (80–95)	85 (80–90)	86 (80–90)
Antithrombotics use, %	58 (37.2)	18 (31.6)	29 (35.4)	37 (38.9)
Onset-to-MRI time, day	2 (1-2)	2 (1-2)	2 (1-2)	2 (1-2)
Onset-to-blood drawing time, day	2 (1-2)	2 (1-2)	2 (1-2)	2 (1-2)
NIHSS	3 (2–5)	3 (2–4.5)	3 (2–5)	3 (2–6)
Fasting glucose, mg/dL	91.8 (85.1–105.6)	92.2 (84.6–116.3)	91.8 (84.6–104.8)	93.6 (86.4–108.0)
Triglycerides, mg/dL	119.0 (89.4–160.2)	113.3 (87.2–149.1)	107.1 (87.4–151.8)	119.5 (87.6–159.3)
HDL, mg/dL	40.8 ± 11.4	41.8 ± 12.9	41.1 ± 11.0	41.8 ± 11.7
LDL, mg/dL	95.0 ± 27.8	93.8 ± 22.2	94.1 ± 26.9	95.7 ± 24.3
Total cholesterol, mg/dL	157.2 ± 36.1	155.9 ± 30.5	155.6 ± 36.3	157.6 ± 34.9
Fibrinogen, mg/dL	296.1 ± 59.4	310.9 ± 61.1^**∗**^	301.2 ± 65.3	295.8 ± 58.1
Cav-1, ng/mL	5.62 ± 2.63	4.74 ± 2.26^**∗∗**^	5.71 ± 2.77	5.65 ± 2.58

CMBs, cerebral microbleeds; SLIs, silent lacunar infarcts; WMHs, white matter hyperintensities; BMI, body mass index; SBP, systolic blood pressure; DBP, diastolic blood pressure; NIHSS, National Institutes of Health Stroke Scale; HDL, high-density lipoprotein; LDL, low-density lipoprotein; Cav-1, caveolin-1.

^*∗*^ *P* < 0.05; ^*∗∗*^ *P* < 0.01 compared with corresponding negative (−) group.

**Table 2:** Logistic regression analysis for association between Cav-1 and cSVD.

Cav-1	CMBs				SLIs				WMHs			
	Model 1	*P*	Model 2	*P*	Model 1	*P*	Model 2	*P*	Model 1	*P*	Model 2	*P*
≤5.25 ng/mL	3.69 (1.78–7.64)	0.00044	4.05 (1.77–9.30)	0.001	1.30 (0.68–2.50)	0.431	1.61 (0.77–3.36)	0.209	0.88 (0.45–1.70)	0.696	0.77 (0.36–1.67)	0.510
>5.25 ng/mL	Reference		Reference		Reference		Reference		Reference		Reference	

Model 1, adjusted for age and sex; Model 2, adjusted for age, sex, hypertension, diabetes mellitus, dyslipidemia, heart disease, BMI, smoking, alcohol intake, SBP, DBP, antithrombotics use, onset-to-MRI time, onset-to-blood drawing time, NIHSS, fasting glucose, triglycerides, HDL, LDL, total cholesterol, and fibrinogen.

CMBs, cerebral microbleeds; SLIs, silent lacunar infarcts; WMHs, white matter hyperintensities.

**Table 3:** Logistic regression analysis for association between Cav-1 and CMBs subgroups.

Cav-1	CMBs location							
	Deep or infratentorial CMBs (*n* = 42)				Strictly lobar CMBs (*n* = 15)			
	Model 1	*P*	Model 2	*P*	Model 1	*P*	Model 2	*P*
≤5.25 ng/mL	3.54 (1.58–7.92)	0.002	4.04 (1.59–10.25)	0.003	1.85 (0.60–5.69)	0.286	1.55 (0.43–5.60)	0.501
>5.25 ng/mL	Reference		Reference		Reference		Reference	

Cav-1	CMBs number							
	Multiple CMBs (*n* = 35)				Single CMBs (*n* = 22)			
	Model 1	*P*	Model 2	*P*	Model 1	*P*	Model 2	*P*

≤5.25 ng/mL	2.70 (1.18–6.17)	0.018	3.18 (1.16–8.72)	0.025	2.72 (0.99–7.45)	0.052	3.03 (0.95–9.72)	0.062
>5.25 ng/mL	Reference		Reference		Reference		Reference	

Model 1, adjusted for age and sex; Model 2, adjusted for age, sex, hypertension, diabetes mellitus, dyslipidemia, heart disease, BMI, smoking, alcohol intake, SBP, DBP, antithrombotics use, onset-to-MRI time, onset-to-blood drawing time, NIHSS, fasting glucose, triglycerides, HDL, LDL, total cholesterol, and fibrinogen.

CMBs, cerebral microbleeds.
